# Can social capital motivate individuals to continue exercising? the case of group extreme conditioning program training

**DOI:** 10.3389/fspor.2026.1792083

**Published:** 2026-04-17

**Authors:** Apostolia Ntovoli, Georgia Stavropoulou, Garyfallos Anagnostou, Panagiotis Papadopoulos, Athanasia Zourladani, Constantinos Giaginis, Kostas Alexandris

**Affiliations:** 1 School of Health Sciences, Department of Life and Health Sciences, Frederick University, Limassol, Cyprus; 2 Laboratory of Management of Sports, Recreation, and Tourism, School of Physical Education and Sport Science, Aristotle University of Thessaloniki, Thessaloniki, Greece; 3 Department of Food Science & Nutrition, School of Environment, University of the Aegean, Thessaloniki, Greece

**Keywords:** exercise intention, exercise motivation, group extreme conditioning program training, self-determination theory (SDT), social capital

## Abstract

**Introduction:**

Social capital is an important concept for individual and societal well-being today. This study aimed to test the relationships among social capital, exercise motivation, and intention to exercise, testing at the same time whether motivation mediates the relationship between social capital and intention to continue exercising. The study was conducted in the context of a group extreme conditioning program training, which has been distinct for its community image and committed member network.

**Methods:**

Five hundred and ninety questionnaires (*N* = 590) were collected, with an online questionnaire, from adult group extreme conditioning program training exercisers. Social capital was measured with nine items, which correspond to the three dimensions: network, trust, and reciprocity. Motivation was measured with twelve items, based on the self-determination theory.

**Results:**

The results indicated that social capital was an antecedent of both intrinsic and extrinsic motivation, but also influenced exercise intention both directly and indirectly, through its influence on motivation.

**Conclusion:**

The trust and reciprocity dimensions of social capital were the most influential ones, while the network dimension had only an indirect relationship with intention, through motivation. The theoretical and applied implications of these results are discussed.

## 1 Introduction

It is well documented today that the development of social capital in leisure contexts is associated with positive physical and psychological outcomes for individuals, and collectively it can contribute to healthy societies (1–4). Lin et al. (5) defined social capital as “investment in social relationships,” which can bring to individuals' “benefits” or “assets” that can be used both in individual and societal levels. These assets relate to social relationships, trust among the members of social networks, and reciprocity, which describes members' willingness for mutual help (6). Group sport and exercise contexts provide a favorable environment for social capital development, since sport involvement creates opportunities for sport participants to become members of social networks, build relationships and trust, and exchange common benefits, all of which are important elements of social capital (7–9),, addressing at the same time several of the Sustainable Development Goals (61) such as SDG3 “SDG 3: ensure healthy lives and promote well-being for all at all ages” and SDG16 “SDG 16: promote peaceful and inclusive societies”.

Empirical research on the positive psychological outcomes of social capital in the context of sports is still limited (9–11). In this paper, we used the Self-determination theory of motivation (12), aiming to examine the role of social capital on the development of exercise motivation, considering at the same time the intrinsic vs. extrinsic paradigm. Previous research on sport motivation has shown that intrinsic motives are the most powerful ones in an individual's decision to start taking part in sports, but also to continue engagement (13–15). However, there is also evidence that some external motives can be internalized by exercisers and finally act as intrinsic motivators (14, 16, 17). It is therefore of theoretical and practical importance to explore whether social capital is one of the factors that lead to increased motivation levels. There has been no published research so far to test the relationship between social capital and motivation, as defined by the self-determination theory, in the context of sport and exercise.

In this paper, we used the group extreme conditioning program training as the context of the study. Group extreme conditioning program training has been shown to be a successful fitness practice recently (18, 19). It has been argued that group extreme conditioning program training is not just a fitness program. It has a strong identity with committed followers, since it has a distinct cultural formation, which is described by social networks, community values, and strong ethics among its members (20, 21). In this line, the group training format promotes social interaction, social support, trust, and reciprocity, contributing to the development of a committed participant base (22–25).

The purpose of this study is therefore to explore if this committed participant base in the group extreme conditioning program training relates to the development of social capital and its influence on members' motivation. The present paper makes a significant contribution to existing research by empirically examining, for the first time, the relationship between social capital and exercise motivation within group fitness contexts through the lens of Self-Determination Theory (SDT). Previous SDT-informed studies have shown that psychological need satisfaction and autonomy-supportive environments are key predictors of sustained motivation and physical activity engagement (24); (62). This is further supported by evidence indicating that SDT principles effectively enhance motivation quality in structured group-based exercise settings (63). Research on the psychosocial outcomes of social capital remains limited (21, 22), making this integrative approach particularly valuable. The findings also have practical implications for the development of group fitness programs and business models—such as those used in group extreme conditioning program training—that leverage social capital to enhance need satisfaction, intrinsic motivation, and long-term exercise adherence.

## 2 Theoretical background

### 2.1 Social capital background

Lin (26) (p.3) defined social capital as the “investment in social relations with expected returns in the marketplace”. These returns or “benefits” are, according to Putnam (27), conceptualized as three core elements: network, trust, and reciprocity, and can be used by members of social networks at both the individual and societal levels (28, 29). Networks, as the core element of social capital, describe the social relationships and interactions among members of social groups. These networks can promote trust among members, access new ideas, and connect individuals across different social hierarchies, thereby fostering cooperation and interaction. They can also extend outside of the exercise environment to members' everyday social and professional lives. Trust is developed when the members of exercise groups develop interpersonal relationships and know each other well (30). Trust facilitates social interaction, communication, and cooperation (24, 31, 32). Finally, reciprocity is developed when the members of social networks share and adopt social norms, values, and ethical codes, expressing their willingness to help each other achieve common goals (33, 34). All three elements of social capital can be particularly applicable in the context of group extreme conditioning program training. Social networks are facilitated because of the group type of training and the common exercise goals, their physically demanding nature, but especially the community approach, which has been part of the positioning strategy for marketing the programs (22, 23, 25). In this line, the members of the extreme conditioning program training networks interact, get to know, and help each other during exercise on achieving individual and group fitness-related goals (22, 23, 25). This willingness to help is further strengthened by the demanding physical nature of training, the common values that are developed in relation to the promotion of an active lifestyle, and the identity built that they are “group extreme conditioning program training members”. Zhou et al. (9) argued that empirical research on the positive psychological outcomes of social capital is still limited in the context of sports. The same authors also argued that the process through which social capital positively influences communities in a long-term perspective is still to be better understood.

### 2.2 The self-determination theory

Motivation refers to the driving force that initiates, directs, and sustains human behaviour (64). According to the Self-Determination Theory (http://www.selfdeterminationtheory.org/theory/), motivation is categorized into three main regulatory styles, as an individual moves from the stages of amotivation, to extrinsic motivation (control orientation) and finally to intrinsic motivation (autonomy orientation), in which individuals are fully self-regulated and are motivated by internal factors (35–37).

Individuals who are intrinsically motivated are engaged in a behavior for its own sake, aiming to have fun, enjoyment, and satisfaction, without expecting any external rewards or pressures (38–40). It is well documented today that intrinsic motivation plays the most influential role in individuals' decisions to engage in exercise behavior and develop exercise adherence (13, 15). Intrinsic motivation leads to several positive behavioral outcomes, such as exercise adherence, intensity and frequency of exercise, trust, learning, and skill acquisition (41, 42). Exercise adherence has been traditionally a common problem. Dropout rates among fitness participants have been reported to be around 75% in the first 3 months of practice, falling to 50% when they reach 6 months of practice (43–45). On the other hand, extrinsic motivation refers to the type of motivation that arises outside of an individual, leading to engaging in behaviors to achieve external tangible or intangible rewards, such as money, praise, recognition, or even avoiding punishment and pressure. It represents, therefore, less self-determined behavior (37). Extrinsic motivation has been further categorized into three sub-dimensions: introjected, identified, and integrated regulation.

Introjected regulation refers to the least autonomous motives, since individuals are internally pressed and feel obliged to act and engage in a specific behavior. In this line, individuals are driven by rewards, social recognition and acceptance, social norms, and even trying to avoid punishment and feelings of guilt and shame (46). Introjected reasons for engaging in a sport activity are usually in compliance with “should” and “must” (47). The next dimension of extrinsic motivation is identified as regulation, in which an individual consciously identifies with the importance of the behaviour, understands the value of engaging because it can help achieving her/his goals. As a result, she/he experiences a relatively high degree of volition or willingness to act and self-directed engagement in the activity (37). Finally, integrated regulation is the most autonomous type of extrinsic motivation. In this case, individual beliefs and values are aligned and used to justify engagement in a specific activity. The reasons for engagement are fully internalized since they are important and fit with a person's personal values and identity. In exercise settings, integrated regulation can be related to the achievement of physical goals, which, during the time, can be internalized, since individuals align with health-related values (14, 48, 49). Still, individuals do not necessarily expect to experience excitement and pleasure during exercise, but they see engagement as important for their physical and psychological health (37). The last stage in the process is amotivation. Individuals in this stage have no reason to continue acting or engaging in behavior; they are likely to drop out or underperform. In a sporting context, amotivated individuals are those who will eventually stop exercising (50). It is well documented today that the different regulatory styles of motivation lead to different outcomes (46, 51). Intrinsic motivation and the most self-regulated type of extrinsic motivation led to increased exercise adherence and involvement. The literature on sport and exercise motivation is extensive; it is not the objective of the paper to provide a detailed review of this literature. A systematic review of the exercise motivation literature can be found in the studies of Standage and Ryan (52), Manninen et al. (53), and Clancy et al. (54).

Based on the above discussion, the objectives of the paper were to test:

1. The relationships between social capital (network, trust, and reciprocity) and intention to continue exercising.
2. The relationships between social capital (network, trust, and reciprocity) and motivation to exercise (intrinsic, extrinsic, and amotivation).
3. Whether motivation mediates the relationship between social capital and intention to continue exercising.

## 3 Materials and research method

### 3.1 Procedure

Following a cross-sectional design, the data were collected with an online survey among exercisers in group extreme conditioning program training activities in Greece. The e-questionnaire was distributed through social media of the research group and relevant blocks (Facebook, LinkedIn, and blogs) to adults (>18 years old), who were exercisers in the group's extreme conditioning program training. Informed consent was obtained from all participants before completing the questionnaire. The research design was approved by the ethics committee of the educational institution (approval code: 286/2025). Five hundred and ninety questionnaires were collected. Addressing the limitations of the research design, it must be noted that the sampling method was a non-probability one. Considering the convenience nature of the sample, the results cannot be considered representative of the study population. Generalizations, therefore, should be made with caution. However, this sampling method was the most effective one in achieving the sample size required to test the theoretical model of the study. This sample size (*N* = 590) was adequately powered to detect small indirect effects in mediation analyses (55).

### 3.2 Participants

Based on the descriptive statistics, the sample consisted of 41.9% men, 58% women, and 0.2% identifying as other. Most participants were single (67.2%), followed by married (26.3%) and divorced (6.5%). Regarding education, the majority hold a bachelor's degree (35.3%) or a master's degree (20.9%), secondary school (31.1%), vocational training (7.8%), or a technological educational institute (4.8%). The average age was 26.9 (SD = 7.1). The young profile of the sample is justified by the type of exercise activity.

### 3.3 Instruments

Social capital was measured with nine items, which correspond to the three dimensions of social capital, as proposed by Putnam (27): network (3 items, *α* = .95), trust (3 items, *α* = .88), and reciprocity (3 items, *α* = .89). Special reference should be made to the scale of Zhou et al. (9), who, based on these three dimensions, developed a nine-item sport social capital scale for participants in sport events. Motivation was measured with 15 items (65, https://selfdeterminationtheory.org/self-regulation-questionnaires) covering the Intrinsic Dimension (3 items, *α* = 89), Extrinsic (9 items, *α* = .92), and Amotivation (3 items, *α* = .85). This scale was previously used in the same cultural environment and was shown to be valid and reliable (56). Finally, intentions to continue participating were measured with three items, as has been typically used in several studies (56–59).

### 3.4 Data analysis

The analysis was made with JASP software (via R). The dataset was examined for missing values before conducting the analyses. The inspection indicated that there were no missing responses across the study variables; thus, no imputation procedures were required, and all 590 participants were included in the analysis. Initially, descriptive statistics were used to better describe the anthropometric characteristics and the variables under investigation, i.e., Social Capital, Motivation, and Intention. A mediation analysis was conducted to examine the direct and indirect effects of Social Capital, which is represented by its three dimensions (Trust**,** Reciprocity, and Network), on Intention, through the mediating roles of Motivations and especially Intrinsic Motivation, Amotivation, and Extrinsic Motivation. The analysis was performed using the maximum likelihood (ML) estimation method. Direct effects represent the influence of each social capital dimension on intention independent of the mediators, whereas indirect effects capture the extent to which this relationship operates through the motivational variables. The Delta method was applied to compute standard errors, and normal theory-based confidence intervals (95%) were used to assess the precision of the estimates. Statistical significance was determined using *z*-values and *p*-values, with *p* < .05 considered significant. The total effects were calculated as the sum of the direct and indirect effects for each predictor.

## 4 Results

Descriptive statistics in the Social Capital, Motivation, and Intention scales indicated that participants reported relatively high levels of Intrinsic Motivation (*M* = 5.27, *SD* = 1.62) and Trust (*M* = 4.92, *SD* = 1.68), followed by Reciprocity (*M* = 4.90, *SD* = 1.70) and Network (*M* = 4.81, *SD* = 1.66). Extrinsic Motivation showed a moderate mean score (*M* = 3.98, *SD* = 1.70), while Intention was also relatively high (*M* = 4.55, *SD* = 1.73). The lowest mean value was observed for Amotivation (*M* = 1.89, *SD* = 1.32). This information is shown in Table 1.

**Table 1:** Descriptive statistics for social capital (trust, reciprocity, network), motivation (extrinsic motivation, intrinsic motivation, amotivation), and intention.

Statistics	Trust	Reciprocity	Network	Extrinsic Motivation	Intrinsic Motivation	Amotivation	Intention
Mean	4.92	4.90	4.81	3.98	5.27	1.89	4.55
Std. Deviation	1.68	1.70	1.66	1.70	1.62	1.32	1.73
Skewness	−.57	−.54	−.50	.08	−.76	1.98	−.23
Std. Error of Skewness	.10	.10	.10	.10	.10	.10	.10
Kurtosis	−.55	−.73	−.80	−.98	−.52	3.69	−1.13
Std. Error of Kurtosis	.20	.20	.20	.20	.20	.20	.20
Minimum	1	1	1	1	1	1	1
Maximum	7	7	7	7	7	7	7

Correlation analysis was used to investigate the relationships between Social Capital (Trust, Reciprocity, Network) and Intention. The results showed that Trust, Reciprocity, and Network were all positively and significantly correlated with Intention. Specifically, trust was moderately to strongly correlated (*r* = .55, *p* < .001), indicating that higher levels of trust are associated with higher levels of intention. Similarly, reciprocity demonstrated a slightly stronger positive relationship (*r* = .59, *p* < .001). Network also shows a strong positive correlation (*r* = .55, *p* < .001), meaning that stronger or more extensive networks are linked to higher levels of intention. The results are represented in Table 2.

**Table 2:** Correlations for social capital (trust, reciprocity, network) and intention*.*

Construct	Trust	Reciprocity	Network
Intention	.55*	.59*	.55*

* *p* *<* *.001.*

Similarly, Pearson Correlation was used to explore correlations between Social Capital (Trust, Reciprocity, Network) and Motivation (Extrinsic Motivation, Intrinsic Motivation, Amotivation). Extrinsic motivation showed small to moderate but statistically significant positive correlations with trust (*r* = .26, *p* < .001), reciprocity (*r* = .26, *p* < .001), and network (*r* = .34, *p* < .001). Intrinsic motivation exhibited stronger positive and significant correlations with trust (*r* = .49, *p* < .001), reciprocity (*r* = .54, *p* < .001), and network (*r* = .49, *p* < .001). In contrast, Amotivation showed very weak and non-significant correlations with trust, reciprocity, and network, implying that a lack of motivation is largely unrelated to these social capital dimensions (Table 3).

**Table 3:** Correlations for social capital (trust, reciprocity, network) and motivation (extrinsic motivation, intrinsic motivation and amotivation).

Dimensions	Trust	Reciprocity	Network
Extrinsic Motivation	.26*	.26*	.34*
Intrinsic Motivation	.49*	.54*	.49*
Amotivation	.01 ns	.02 ns	.07 ns

* *p* *<* .001, ns, non-significant.

### 4.1 Mediation analysis

**Figure 1:**
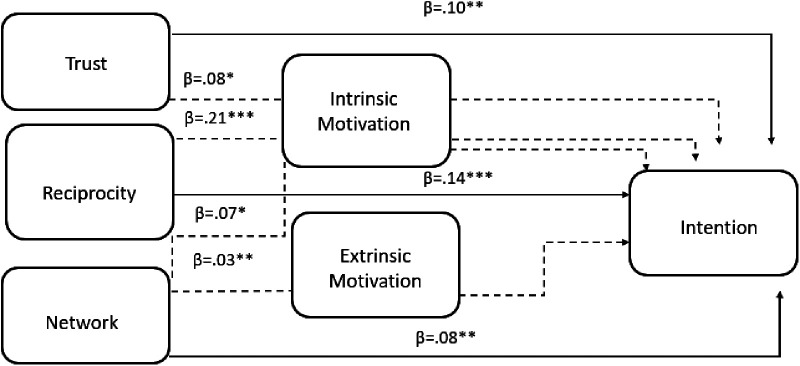
Direct and indirect effects from social capital (trust, reciprocity, network) to intention via motivation (intrinsic and extrinsic). *Note^a^*: *p < .05, **p < .01, ***p < .001, ns = non-significant. *Note*^b^: Dashed lines indicate indirect effects, whereas solid lines indicate direct effects.

A mediation analysis was conducted to examine how the three dimensions of Social Capital, Trust, Reciprocity, and Network influence Intention, both directly and indirectly, through three types of Motivation: Intrinsic Motivation, Amotivation, and Extrinsic Motivation (Figure 1). The model demonstrated good fit, *χ*² (4) = 20.357, *p* < .001 CFI = .98, TLI = .93, RMSEA = .08, 90% CI [.05,.12], SRMR = .05.

The results revealed that trust had a significant positive direct effect on Intention [*β* = .10, *p* < .05, 95% CI (.02,.18)], indicating that individuals with higher trust levels tend to report stronger intentions. Among the indirect pathways, Intrinsic Motivation significantly mediated the effect of Trust on Intention [*β* = .08, *p* < .05, 95% CI (.01,.14)]. This suggests that Trust enhances Intention partly by increasing individuals' intrinsic motivation. The indirect effects through Amotivation (*β* = .01, *p* = .249) and Extrinsic Motivation (*β* = .00, *p* = .580) were not significant, indicating no mediation via these two motivation types. The total effect of Trust on Intention was significant (*β* = .19, *p* < .001), confirming that both direct and indirect mechanisms are important. Intrinsic Motivation serves as a partial mediator between Trust and Intention.

Reciprocity also demonstrated a significant direct effect on Intention [*β* = .14, *p* < .001, 95% CI (.06,.22)]. This finding indicates that individuals who experience greater reciprocity within their social relationships are more likely to form stronger intentions. A strong and significant indirect effect through Intrinsic Motivation was found [*β* = .21, *p* < .001, 95% CI (.14,.27)]. This shows that Reciprocity increases Intention primarily by enhancing Intrinsic Motivation. The indirect effects via Amotivation (*β* = .00, *p* = .666) and Extrinsic Motivation (*β* = -.00, *p* = .929) were non-significant. The total effect of Reciprocity on Intention was highly significant (*β* = .35, *p* < .001), demonstrating that Reciprocity exerts both direct and indirect influences. These results indicate partial mediation. Hence, Intrinsic mediates a substantial part of this relationship.

The direct effect of Network on Intention was positive but marginally non-significant [*β* = .08, *p* = .074, 95% CI (-.01,.16)]. However, the indirect effects reveal important mediation mechanisms. Specifically, the indirect effect through Intrinsic Motivation [*β* = .07, *p* < .01, 95% CI (.01,.14)] and through Extrinsic Motivation [*β* = .03, *p* < .05, 95% CI (.01,.05)] were both significant. This means that a strong social network contributes to Intention by increasing both intrinsic and extrinsic forms of motivation. The indirect path through Amotivation (*β* = −.01, *p* < .01) was marginally non-significant. The total effect of Network on Intention was significant (*β* = .17, *p* < .01), indicating that the influence of Network on Intention operates primarily through motivational mechanisms, especially intrinsic and extrinsic motivation. The total indirect effects were significant for Trust (*β* = .09, *p* < .05), Reciprocity (*β* = .21, *p* < .001), and Network (*β* = .09, *p* < .05), supporting the overall mediating role of motivational factors in the Social Capital-Intention link. Direct and indirect paths are represented in Tables 4, 5.

**Table 4:** Direct paths and standardized regression coefficients (β).

Predictor	Outcome	Estimate (*β*)	SE	z	P	95% CI
Trust	Intention	.10	.04	2.38	<.01	(.02,.18)
Reciprocity	Intention	.14	.04	3.27	<.001	(.06,.22)
Network	Intention	.08	.04	1.79	<.01	(−.01,.16)

**Table 5:** Indirect paths and regression coefficients (β).

Predictor	Mediator	Outcome	Estimate (β)	SE	Z	p	95% CI
Trust	Intrinsic Motivation	Intention	.08	.03	2.30	<.05	(.01,.14)
Trust	Amotivation	Intention	.01	.01	1.15	.25	(−.00,.02)
Trust	Extrinsic Motivation	Intention	.00	.01	.55	.58	(−.01,.02)
Reciprocity	Intrinsic Motivation	Intention	.21	.04	5.87	<.001	(.14,.27)
Reciprocity	Amotivation	Intention	.00	.01	.43	.666	(−.01,.01)
Reciprocity	Extrinsic Motivation	Intention	−5.286 × 10^−4^	.01	−.09	.929	(−.01,.01)
Network	Intrinsic Motivation	Intention	.07	.03	2.18	<.05	(.01,.14)
Network	Amotivation	Intention	−.01	.01	−1.73	.084	(−.02,.00)
Network	Extrinsic Motivation	Intention	.03	.01	2.56	<.01	(.01,.05)

## 5 Discussion

### 5.1 The relationship between social capital and intention

This study aimed to test the relationships among social capital, motivation, and intentions, in the context of group extreme conditioning program training activities, aiming at the same time to examine if motivation acts as a mediator of the relationship between social capital and intentions. Previous SDT-informed research has demonstrated that satisfaction of the basic psychological needs, together with autonomy-supportive social environments, reliably predicts sustained motivation and continued engagement in physical activity (62, 66). However, the relationships between social capital, motivation, and intentions under the STD framework have not been examined in an integrated model in the context of group exercise.

The results first confirmed the positive relationship between social capital and intention to participate. Social capital is, therefore, a significant antecedent of exercise commitment; it might be an explanation for the commitment of group extreme conditioning program training members' networks, as has been previously reported (22, 23, 25). Reciprocity was the dimension with the strongest influence on intention. Reciprocity is developed when members of social groups express willingness to help each other and exchange benefits or assets (27). It is also characterized by members' willingness to express new ideas, share common values, and cultivate a common culture (34).

The extreme group training environment naturally fosters reciprocity, as members support each other through shared challenges that satisfy key psychological needs and enhance autonomous motivation (63, 66). Consequently, this type of exercise marketing and delivery operates as effective community-based models that build group identity and promote adherence, supported by evidence of higher social capital and belongingness compared with traditional exercise programs (21, 67). Trust also emerged as a key predictor of continued participation, consistent with SDT's emphasis on relatedness as a driver of internalization and sustained engagement (63, 66). This trust often extends beyond the exercise environment, reinforcing strong interpersonal bonds within extreme exercise group communities (21) and aligning with broader social-capital theory highlighting trust and reciprocity as foundations for coordinated action (27).

Offering mutual help in the highly physically and psychologically demanding environment of extreme group exercise (60) is a good practice for the members to achieve personal and mutual exercise goals. Exercise leaders and instructors can also play an important role in the development of an exercise environment that promotes social interaction, interpersonal relationships, and trust among members. The final dimension of social capital—networks—offered a positive but marginally non-significant contribution to the prediction of exercise intention. This finding indicates that exercising in a group setting is not enough for the development of exercise commitment if trust and reciprocity are not present. Once again, it can be argued that the successful community positioning of these types of programs played an important role in their development (20, 21).

### 5.2 Social capital, motivation, and intention

As previously noted, intrinsic motivation has been shown to be the most important predictor of exercise participation and adherence (13, 15); furthermore, there might be cases where introjected regulation can be internalized and act as intrinsic motivation (16, 17). It has, therefore, theoretical and applied value to examine whether social capital is one of the antecedents of exercise motivation. The results indicated that all three dimensions of social capital—reciprocity, trust, and network—had significant and strong relationships with intrinsic motivation, significant and weak to moderate with extrinsic motivation, and no significant relationships with amotivation. These findings contribute to advancing theoretical understanding of exercise motivation by identifying, for the first time, the specific exercise conditions under which both intrinsic and extrinsic motivation can be enhanced. Exercise environments that encourage the exchange of mutual benefits, foster trust among participants, and promote a shared identity grounded in common values and culture appear particularly effective in strengthening motivated behavior. Such conditions are central to the social dynamics observed in training communities and help explain the notably high levels of commitment reported among these participants (22, 23, 25). In these settings, reciprocity, interpersonal trust, and a cohesive social network operate as key motivational drivers that reinforce continued participation and long-term exercise adherence. The positive correlation between social capital and extrinsic motivation confirms what has been previously reported, that there are cases where certain extrinsic motives (introjected) can be internalized over time, when individuals identify with behaviors that have specific benefits for them, such as health ones in the case of exercise (16, 17). Subsequently, the development of social capital in exercise settings can increase both intrinsic and extrinsic exercise motivation.

The final objective of this study was to examine whether different dimensions of motivation mediate the relationship between social capital and intention to continue exercising. Consistent with prior SDT-based findings showing that social environments shape motivational quality through need-supportive mechanisms (62, 66), the results revealed that intrinsic motivation significantly, though only partially, mediated the associations between both trust and reciprocity and exercise intention. Thus, trust and reciprocity exerted both direct effects on intention and indirect effects through their capacity to enhance intrinsic motivation. These findings reinforce previous evidence demonstrating that positive social processes—such as interpersonal support, cooperation, and value sharing—serve as important antecedents of intrinsic motivation and exercise commitment in group-based physical activity contexts (52, 63).

In contrast, the relationship between the network dimension of social capital and intention was fully mediated by both intrinsic and extrinsic motivation. This aligns with research indicating that structural social ties influence behavior primarily when they contribute to the internalization of motivational regulations (16, 17). The present findings, therefore, suggest that the mere presence of social networks within exercise environments is insufficient to promote adherence; rather, their impact becomes meaningful only when they enhance the quality of exercisers' motivation. This observation supports previous evidence that motivational processes constitute the central pathway through which social factors promote sustained engagement in physical activity (66, 68).

These findings have practical implications. First, they explain the successful business model of group extreme conditioning program training, both from a marketing and delivery perspective (20). Using community image as the main marketing and positioning strategy and delivering it in an environment that promotes common values, culture, and ethics were important factors for this type of training's global success (21). Group exercise is one of the factors that can influence exercise motivation and intention, but it is not the determining one. Instead, exercise program developers and leaders should create environments that foster the development of reciprocity and trust among the exercise members. Group exercise programs with a strong culture that help participants to identify with their brand attributes and express their personal identity can strengthen participants' intrinsic motivation. Furthermore, social networks should be extended outside of the exercise environment, with the development of trust among members that helps them to exchange information and help each other in their social lives.

### 5.3 Study limitations and future research

The study followed a cross-sectional design, which means that causal relationships among the study's variables cannot be supported. A longitudinal approach is the proper method that can be used for testing causal relationships and examining the stability of behavior (e.g., exercise commitment). Future studies that employ longitudinal or repeated-measures designs would also help determine whether the influences of reciprocity, trust, and networks become stronger or weaker over time, and whether specific social interactions accelerate the internalization of extrinsic motives into more self-endorsed forms. Such work would also enable researchers to test potential bidirectional relationships, as existing SDT scholarship indicates that motivation may shape social relationships as much as social environments shape motivation. As previously noted, group extreme conditioning program training is a type of exercise with a specific brand, identity, and culture. It is interesting from a theoretical and applied perspective to examine the relationships among social capital, motivation, and commitment in other exercise settings and/or programs with similar or different images, such as triathlon events, running marathons, skateboarders, Spartan races, etc. Testing these relationships by controlling demographic variables, and especially age groups, is also a suggestion for future research. Using, for example, the age cohort theory can offer new insights into how social capital is developed and influences exercise behavior among different target groups. Cross-cultural research also represents an important future direction. Prior work applying SDT in physical education settings underscores the relevance of cultural context for motivational processes and needs satisfaction. Comparative studies across cultures could reveal whether the structure and impact of social capital differ in societies, and whether cultural norms shape how trust, reciprocity, and group identity operate within exercise communities. Such research would enhance the global applicability of the proposed theoretical model. A final promising future research direction concerns the evolving role of digital and hybrid exercise environments. SDT-informed research emphasizes the importance of interpersonal support for sustained exercise behavior. As virtual and hybrid fitness programs continue to expand, future scholarship should investigate how social capital is formed and experienced online, whether virtual trust and reciprocity exert the same motivational influence as in-person interactions, and which digital design contexts facilitate the satisfaction of psychological needs for autonomy, competence, and relatedness.

## 6 Conclusion

This study offers the first empirical evidence demonstrating the pivotal role of social capital in shaping exercise motivation and exercise commitment within group-based fitness contexts. The findings indicate that social capital functions as a significant antecedent of both intrinsic and extrinsic motivation and further influences exercise intention through both direct effects and indirect pathways mediated by motivational processes. Among its dimensions, trust and reciprocity emerged as the most influential predictors of intention, whereas the network dimension contributed only indirectly, operating primarily through its impact on motivational regulation. These outcomes help explain the rapid expansion and sustained popularity of group extreme conditioning program training, which relies heavily on a community-oriented model that cultivates interpersonal communication, cooperation, trust, and reciprocal exchanges among members. These results suggest that exercising in a group is not enough; rather, environments must be intentionally designed to foster reciprocity, trust, and meaningful identity-building. For practitioners, this provides valuable guidance: successful models like group extreme conditioning programs thrive because they leverage strong community branding, shared values, and supportive interpersonal dynamics. Exercise leaders who cultivate these conditions can enhance both intrinsic and extrinsic motivation, ultimately promoting long-term adherence. Extending social networks beyond the workout setting—through communication, shared activities, and mutual support—further reinforces these benefits. Accordingly, programs that integrate social capital–building strategies into both marketing and delivery stand to improve participation, retention, and overall exercise commitment.

Finally, the findings highlight the broader societal value of group-based exercise by aligning with several Sustainable Development Goals (UN, 2024), particularly SDG 16, which emphasizes the promotion of peaceful and inclusive societies. These programs, as a form of community-based group exercise, have the potential to foster friendship, mutual understanding, and social cohesion by bringing together individuals from diverse cultural and social backgrounds and facilitating meaningful interaction through shared physical activity. In this way, group exercise programs do not merely support physical health but also contribute to social well-being and community building.

## Data Availability

The datasets presented in this article are not readily available because we stated in the consent form and the ethics committee that the data is confidential. Requests to access the datasets should be directed to antovoli@phed.auth.gr.
